# Detection of Structural Changes in G-Quadruplex-Forming DNA Oligonucleotides via DNA Methylation Based on Luminol Chemiluminescence Catalyzed by Myoglobin

**DOI:** 10.3390/bios16010001

**Published:** 2025-12-19

**Authors:** Shintaro Inaba, Haruka Kawai, Mizuki Tomizawa, Daimei Miura, Kaori Tsukakoshi, Kazunori Ikebukuro

**Affiliations:** 1 Department of Biotechnology and Life Science, Graduate School of Engineering, Tokyo University of Agriculture and Technology, 2-24-16 Naka-cho, Koganei 184-8588, Tokyo, Japan; 2 SilCreTech Co., Ltd., 3-59-10 Kameido, Koto-ku 136-0071, Tokyo, Japan; 3 Institute of Global Innovation Research, Tokyo University of Agriculture and Technology, 3-8-1 Harumi-cho, Fuchu 183-8538, Tokyo, Japan; 4 Department of Chemistry, Faculty of Science, Tokyo University of Science, 1-3 Kagurazaka, Shinjuku-ku 162-8601, Tokyo, Japan

**Keywords:** CpG methylation, G-quadruplex, myoglobin, biosensor

## Abstract

A novel, label-free chemiluminescence sensing platform for CpG methylation was developed, leveraging the G-quadruplex (G4) structural sensitivity of G4–protein interactions to eliminate bisulfite conversion. This sensing system is based on the enhancement of luminol chemiluminescence generated from myoglobin upon binding to the G4-forming DNA. At the core of this biosensor is the G4-structure-dependent modulation of the peroxidase-like activity generating luminol chemiluminescence of myoglobin. The structural change by CpG methylation within the G4-forming sequence of the B cell lymphoma 2 (BCL2) gene promoter altered its binding to myoglobin, transducing the methylation state into a measurable signal catalyzed by myoglobin. This principle was validated in a practical assay using immobilized probes to capture the target DNA for methylation analysis. This system demonstrated the capability to distinguish methylation differences of 50% when the target DNA concentration was over 25 nM. Versatility was further confirmed using the sequence from the dopamine receptor D2 (DRD2) gene promoter, where the methylation similarly induced distinct topological and functional changes. This is the first study to directly link the epigenetic state of a G4-forming DNA sequence to a protein-mediated enzymatic output, offering a framework for simple, rapid, and highly adaptable biosensors for research and clinical applications.

## 1. Introduction

DNA methylation, which involves the addition of a methyl group to the 5-position of a cytosine residue, is an essential epigenetic modification [1] that plays an important role in gene regulation processes such as X-chromosome inactivation [2], genomic imprinting [3], and the maintenance of genome stability [4]. In normal cellular functions in humans, this modification occurs at cytosines within CpG dinucleotides [5]. On the other hand, abnormal methylation, including hypermethylation or hypomethylation, sometimes occurs, which is strongly linked to various diseases such as cancer [6,7,8], congenital disorders [9,10], and psychiatric conditions [11,12,13]. Therefore, CpG methylation is widely recognized as a promising biomarker for diagnosis, and the demand for rapid, reliable, and user-friendly detection methods is growing.

Currently, the most widely used methods for detecting CpG methylation are based on bisulfite conversion [14,15,16]. Although it provides accurate and detailed methylation information, bisulfite conversion not only requires the use of harsh chemicals that can damage the genome but also lacks throughput [17,18]. Non-bisulfite methods, such as those using methylation-sensitive or dependent restriction enzymes [19,20,21,22], antibodies [23,24], or methyl-CpG-binding proteins [25,26], provide simpler workflows. However, these methods are time-consuming, complex, and require costly reagents.

In the human genome, methylation is not randomly distributed, but particularly concentrated in regions called CpG islands, where CpG sites are often clustered. CpG islands are frequently found in gene promoter regions, and ~70% of human gene promoters are estimated to be associated with CpG islands [27]. In addition, CpG islands are enriched in G-quadruplex (G4)-forming sequences [28,29,30]. Furthermore, CpG methylation in G4-forming sequences can alter the G4 topology [31,32,33], thermal stability [33,34], and protein-binding behavior [31]. Such changes in G4 properties induced by CpG methylation are attributed to the CH–π interactions between the methyl group of the methylated cytosine and the adjacent bases [35]. These findings indicate that G4s are not only structural motifs involved in gene regulation but can also serve as potential indicators of the methylation state.

Focusing on methylation-induced changes in the G4 structure, we previously developed methylation detection strategies that utilize the alterations in the initial elongation efficiency of the polymerase chain reaction (PCR) [36,37] and differences in binding affinity with G4 ligands caused by G4 conformational changes induced by CpG methylation [38]. Although our approach effectively detected G4 structural changes induced by CpG methylation, it required either the synthesis of chemically complex ligands or the use of specialized instruments, such as a thermal cycler or heater. These requirements hinder its application in point-of-care diagnostics, which should be simple and cost-effective. Therefore, alternative methods that can leverage information derived from G4 structures while being compatible with simple, affordable, and instrument-free detection platforms are needed.

**Figure 1:**
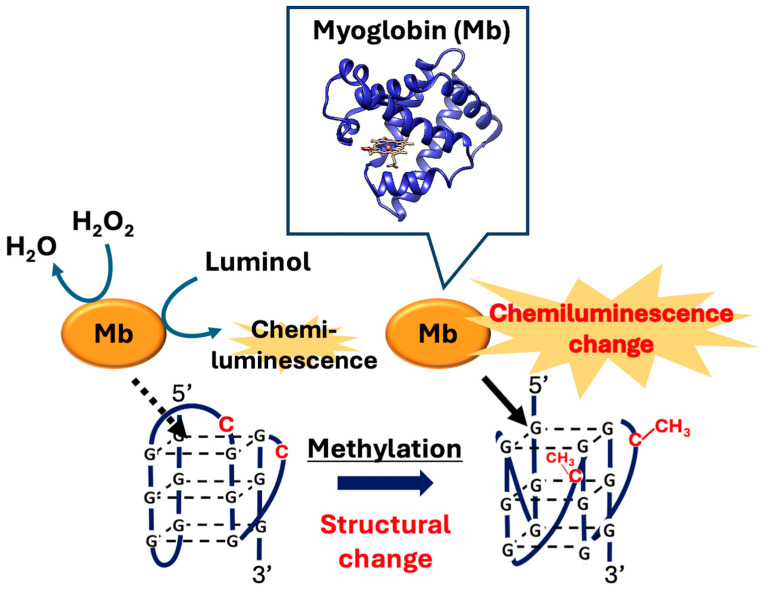
Schematic of CpG methylation detection strategy based on G4 structural changes induced by CpG methylation, which regulates luminol chemiluminescence catalyzed by myoglobin.

Here, we focused on how CpG methylation induces G4 structural changes and how this event can be converted into detectable and quantifiable signals. Myoglobin (Mb), a conjugated protein consisting of a single polypeptide chain related to an iron–porphyrin complex and the heme group [39], exhibits weak peroxidase-like activity [40]. We have demonstrated that G4 structures can bind to myoglobin near the heme and significantly enhance its peroxidase-like activity with increased luminol chemiluminescence [41]. Additionally, we have shown that this enhancement varies depending on the DNA sequence forming the G4 structure and topology. Therefore, we propose a novel signal transduction technology based on G4 structural changes induced by CpG methylation, leading to a difference in myoglobin-derived peroxidase-like activity (Figure 1). By leveraging this intrinsic activity as a direct indicator, the proposed system eliminates the need for external labeling and complex pretreatment steps, thus enabling label-free methylation analysis and providing a rapid and simple analytical platform. Recently, label-free chemiluminescence detection has attracted attention as a promising approach for simple and cost-effective analysis.

To model the oligonucleotides forming the G4 structure, we focused on the sequences of the promoter regions in the B cell lymphoma 2 gene (BCL2) and dopamine receptor D2 gene (DRD2). Methylation in the BCL2 promoter region has been associated with cancer [42,43]. Part of this gene promoter forms the G4 structure, and the structure is changed by methylation [31,33]. Similarly, abnormal methylation in the DRD2 promoter region has been linked to schizophrenia [11]. Methylation in this region is focused on as the diagnostic target. Presently, the clinical diagnosis of schizophrenia relies on patient interviews, which depends on subjective self-reporting, and distinguishing its symptoms from other mental illnesses is challenging [44,45].

In this work, we established a proof of concept for a novel detection strategy by demonstrating that CpG methylation-induced structural changes in G4-forming oligonucleotides can be transduced into measurable signals via myoglobin-catalyzed luminol chemiluminescence.

## 2. Materials and Methods

### 2.1. DNA Oligonucleotides

All oligonucleotides employed in this study were chemically synthesized by Eurofins Genomics (Tokyo, Japan), Tsukuba Oligo Service (Ushiku, Japan), or FASMAC (Atsugi, Japan). Specifically, the methylated DNA sequences were synthesized with 5-methylcytosine at all CpG sites, serving as the 100% methylation samples. The defined methylation levels were controlled by mixing unmethylated (0%) and methylated DNA (100%) samples at a specific molar ratio. The DRD2 sequence (seq) is a DNA fragment derived from the promoter region in DRD2, and BCL2-G4 is a DNA fragment that is part of the promoter region in BCL2. BCL2-87 is an 87-mer DNA fragment comprising a BCL2-G4 sequence. All the oligonucleotide sequences are shown in Table S1. All CpG sites in the methylated DNA sequences were methylated.

### 2.2. Circular Dichroism (CD) Measurement

CD measurements were performed at 25 °C using a spectropolarimeter (J-820, JASCO Corporation, Tokyo, Japan). The spectra of the samples were acquired using a quartz cuvette with a path length of 1 mm. The scanning speed was 50 nm min^−1^, the data acquisition interval was 0.5 nm, and the response time was 4 s. The spectra are shown as the average of five scans. Temperature ramping spectral measurements were performed in 5 °C steps from 5 °C to 95 °C using the spectropolarimeter. All measurements were performed using DNA oligonucleotides at a concentration of 20 µM in 10 mM Tris-HCl supplemented with 100 mM KCl (pH 7.4).

### 2.3. Principal Component Analysis (PCA)

PCA of the CD spectra was performed to estimate the tertiary structures of the BCL2-G4 and DRD2 sequences. A library of 30 reference CD spectra of G4 structures was used to create the PCA model; 22 of the reference CD spectra of the G4-forming sequence library were previously published, and our group obtained eight [46,47]. The CD spectra were preprocessed by normalizing and centering. Hierarchical clustering was conducted using five principal components.

### 2.4. Multivariate Curve Resolution (MCR) with Thermodynamic Model Constraint

The temperature ramping spectral matrix, X, was decomposed into the concentration matrix, C, and pure spectra matrix, S, by MCR with a thermodynamic model constraint. The decomposition procedure is described in detail elsewhere [47]. The number of components and initial concentration, C0, were estimated using PCA and evolving factor analysis. (EFA). X was decomposed into C1 and S using alternating least squares (ALS), C1 was updated to C2 by global fitting with a thermodynamics model, and C2 was applied to the next ALS cycle. This process was iterated until convergence. Computational calculations were performed using Python 3.7.4 with Numpy 1.17.2., lmfit 1.2.2., and pyMCR 0.5.1.

### 2.5. Native Polyacrylamide Gel Electrophoresis (PAGE)

Samples were prepared by mixing the following: 2 µL of 100 μM unmethylated/methylated DRD seq or unmethylated mutant DRD2 seq; 10 µL of 100 mM Tris-HCl containing 1 M KCl (pH 7.4); and 88 µL of Milli-Q water. The mixture was denatured at 95 °C for 10 min, then gradually cooled to 25 °C over 30 min. After loading 5 μL each of the folded oligonucleotides into 20% polyacrylamide gel, electrophoresis was run at 20 mA for 1 h at 25 °C. Afterward, the gel was stained with SYBR^TM^ gold nucleic acid gel stain (Thermo Fisher Scientific, Waltham, MA, USA) at 25 °C for 20 min. The gel image was obtained using a Gel DocTM EZ imager purchased from Bio-Rad (Hercules, CA, USA).

### 2.6. Dot Blotting Assay

The binding between unmethylated/methylated DRD2 seq or BCL2-G4 and myoglobin was investigated in a dot blotting assay. The DNA oligonucleotides were directly biotinylated at the 5′ termini. These oligonucleotides (1 µM) were heated and folded in 10 mM MES containing 100 mM KCl and 10 mM NaCl (pH 6.0) under the same conditions as those described above. Then, 1 µL of myoglobin diluted to varying concentrations with MES buffer containing 0.05% Tween-20 (MES-T) was spotted onto a nitrocellulose membrane and dried. The membrane was blocked with 5% (*w*/*v*) bovine serum albumin in MES-T at 25 °C for 1 h. After washing with MES-T three times, the membrane was incubated with each of the DNA oligonucleotides (100 nM) at 25 °C for 1 h. After washing with MES-T three times, the membrane was incubated with streptavidin alkaline phosphatase (ALP) (Promega, Madison, WI, USA), and chemiluminescence was detected using an Immobilon Western chemiluminescent ALP substrate (Merck Millipore, Burlington, MA, USA) with Image Quant LAS 4000 (GE Healthcare, Chicago, IL, USA).

### 2.7. Detection of CpG Methylation in G4 DNA Using Luminol Chemiluminescence Catalyzed by Myoglobin

The DNA oligonucleotides were directly biotinylated at the 5′ termini. These oligonucleotides (2 µM) were heated and folded in MES buffer under the same conditions as those described above. Then, they were diluted with the same buffer, added to a streptavidin-coated 96-well plate (Thermo Fisher Scientific), and incubated at 25 °C for 1 h. The wells were washed with MES-T and blocked with MES-T containing 4% (*w*/*v*) skim milk at 25 °C for 1 h. After washing, myoglobin was added and incubated at 25 °C for 1 h. Finally, after washing, a BM chemiluminescence ELISA substrate (POD) (Roche Diagnostics, Mannheim, Germany) was added, and the chemiluminescence intensity was measured using a Varioskan Flash (Thermo Fisher Scientific).

### 2.8. Experimental Procedure for Detection of CpG Methylation in Target DNA Captured by a DNA Probe

The 5′-biotinylated 3′-probe targeting the 3′-region of BCL2-87 was heated to 95 °C for 10 min and quenched on ice. The heat-treated 3′-probe was diluted to 200 nM with MES buffer and added to a streptavidin-coated 96-well plate. After incubation at 25 °C for 1 h and washing three times, the blocking buffer was added, and the probe was incubated at 25 °C for 1 h. Finally, the wells were washed. Methylated/unmethylated BCL2-87 or polyT-87 was mixed with the 5′-probe, heated at 95 °C for 10 min, and gradually cooled to 25 °C. Then, they were diluted with either the same buffer or the same buffer spiked with human serum (Cosmo Bio, Tokyo, Japan) to a final concentration of 5%. The solutions were added to the 3′-probe-immobilized plate and incubated overnight at 25 °C. After washing the wells with buffer, 100 μL of 200 nM myoglobin was added, and the solution was incubated at 25 °C for 1 h. After washing, 100 μL of the BM chemiluminescence ELISA substrate (POD) (Roche Diagnostics) was added. The chemiluminescence intensity was measured using a Varioskan Flash (Thermo Fisher Scientific).

### 2.9. Statistical Analyses

Data are presented as the mean ± standard deviation (SD), unless otherwise stated. The statistical analysis for each experiment is described in the figure legends. The numbers of technical and biological replicates for each result are also described in the figure legends. All the statistical analyses were performed using GraphPad Prism version 10.50 for Macintosh (GraphPad Software, San Diego, CA, USA)

The significance of the different results was analyzed using a multiple unpaired *t*-test (two-tailed) or one-way ANOVA, followed by Tukey’s post hoc test.

## 3. Results and Discussion

### 3.1. Investigation of the Interaction Between BCL2-G4 and Myoglobin

First, to investigate the binding interaction between myoglobin and G4 and assess the influence of CpG methylation on this interaction, a dot blotting assay was performed. In this study, we focused on a G-rich sequence from the BCL2 promoter region (BCL2-G4) in chromosome 18 (hg38, chr18:63320132–63332159). This sequence is known to undergo a structural change upon CpG methylation [31,33], a phenomenon we also confirmed in our own experiments using PCA based on CD spectroscopy. Specifically, our analysis indicated that unmethylated BCL2-G4 formed hybrid G4 and altered parallel G4 structures by CpG methylation (Figure S1), which was consistent with the results of previous reports [31].

**Figure 2:**
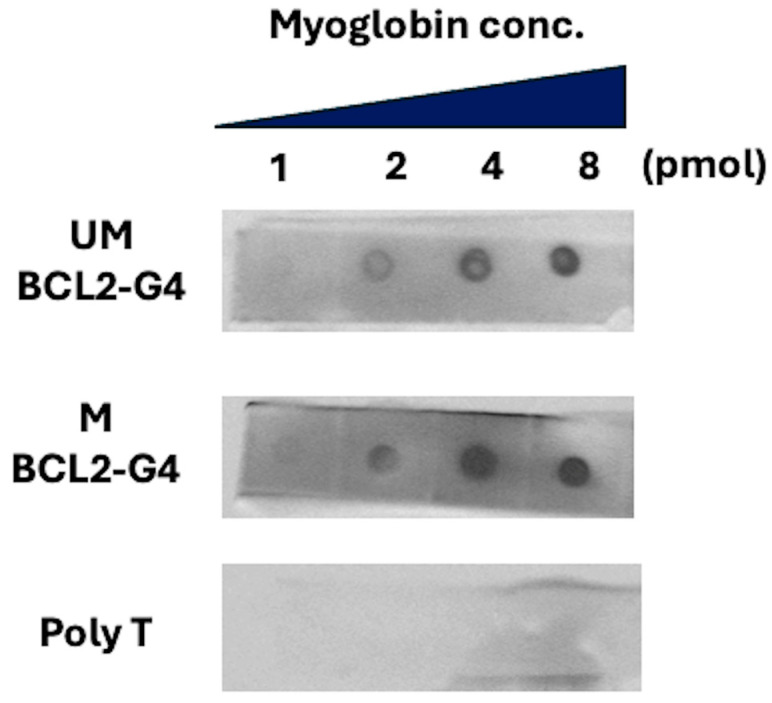
Results of binding ability analysis by dot blotting of 100 nM unmethylated/methylated BCL2-G4 and polyT. PolyT was used as a negative control, as it does not form a G4 structure. UM: unmethylated; M: methylated.

The results are shown in Figure 2. The spot signal intensity increased depending on the immobilized myoglobin concentration, indicating that both unmethylated and methylated BCL2-G4 were bound to myoglobin. However, the spot signal intensities of unmethylated and methylated BCL2-G4 at low myoglobin concentrations differed. The spot signal intensities were analyzed using ImageJ software (version 1.54g), and at myoglobin concentrations of 1 and 2 μM, methylated BCL2-G4 consistently exhibited a higher spot than unmethylated BCL2-G4 (methylated signal intensities: 120, 140 arbitrary units (a.u.); unmethylated signal intensities: 90, 120 a.u., respectively). Therefore, methylated BCL2-G4 bound more strongly to myoglobin than unmethylated BCL2-G4. Furthermore, to distinguish between the effect of the G4 structural change and the direct influence of the methyl base on myoglobin binding, a dot blotting assay was performed using unmethylated and methylated mutant BCL2-G4 sequences. This mutant was designed to be deficient in G4 formation by altering a GGG motif to GTG within BCL2-G4 (Table S1). As a result, at low myoglobin concentrations (1 and 2 μM), both unmethylated and methylated mutant BCL2-G4 produced weaker signals than that of wild-type unmethylated BCL2-G4 and were essentially indistinguishable from each other (Figure S2). These results suggest that the enhanced binding affinity of methylated BCL2-G4 for myoglobin was a consequence of its methylation-induced structural transition from a hybrid to a parallel G4 topology. Our group has reported that many myoglobin aptamers form parallel G4 structures that strongly bind to myoglobin [41]. Taking this into consideration, we propose that the structural change from hybrid G4 to parallel G4 conformation by CpG methylation enhances the ability of the G4 structure to bind to myoglobin.

### 3.2. Detection of CpG Methylation in BCL2-G4 Using Myoglobin

**Figure 3:**
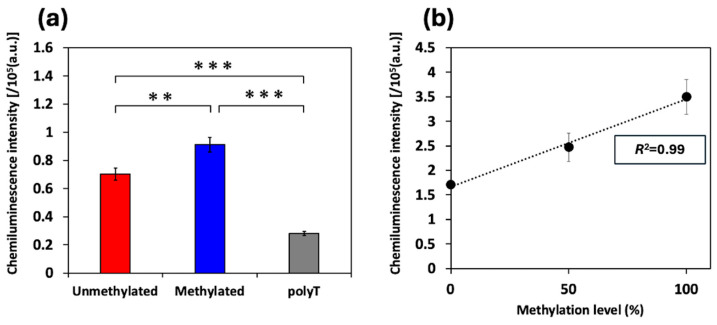
(**a**) Chemiluminescence intensity measurements of 200 nM unmethylated/methylated BCL2-G4 and polyT. (**b**) Calibration curve of chemiluminescence intensity from BCL2-G4. Unmethylated and methylated DNA samples were mixed to prepare samples with varying methylation levels. All plots and bars are shown as the mean ± SD on the graph (*n* = 3). The statistical significance in (**a**) was determined via a one-way ANOVA, followed by Tukey’s multiple comparisons test (**: *p* < 0.01, ***: *p* < 0.001).

Next, G4 DNA methylation was detected on the basis of changes in myoglobin-derived peroxidase-like activity. As shown in Figure 3a, when BCL2-G4 samples at a concentration of 200 nM were used, the chemiluminescence intensity of the methylated BCL2-G4 was significantly higher than that of the unmethylated BCL2-G4. The chemiluminescence intensities of the unmethylated and methylated mutant BCL2-G4 were comparable to that of polyT, as a negative control (Figure S3). Therefore, the structural change induced by CpG methylation was detected using luminol chemiluminescence catalyzed by myoglobin.

To investigate whether the intensity of chemiluminescence catalyzed by myoglobin depends on the methylation level, we prepared samples with varying methylation levels of BCL2-G4. The results show that the chemiluminescence intensity increased with methylation level, which ranged from 0% to 100% (Figure 3b). Thus, we obtained a linear calibration curve (*R*^2^ = 0.99) and estimated the methylation level of the target DNA by drawing a calibration curve. The mean intensity increased from ~1.7 × 10^5^ a.u. (SD = 6.4 × 10^4^ a.u.) for 0% methylation to 3.5 × 10^5^ a.u. (SD = 3.5 × 10^4^ a.u.) for 100% methylation. The average sensitivity was 1.8 × 10^3^ a.u. signal change for 1% methylation. Considering this sensitivity alongside the measurement variability (represented by a pooled SD of ~2.6 × 10^4^ a.u.), our analysis results indicate that this method can distinguish the differences in BCL2-G4 methylation larger than 40% based on the 3σ criterion.

### 3.3. Evaluation of CpG Methylation Detection in Target DNA Captured by a DNA Probe

**Figure 4:**
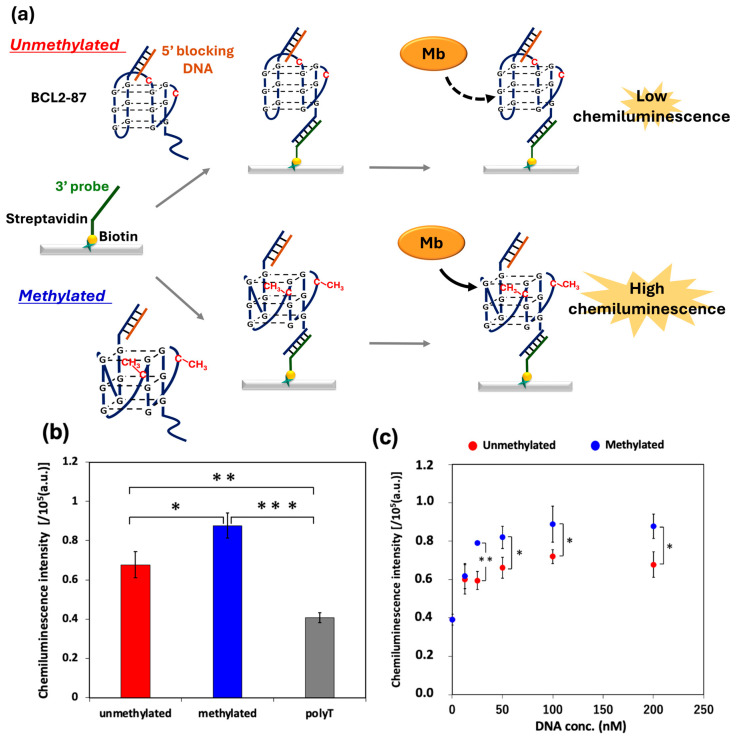
(**a**) Improved CpG methylation detection scheme using myoglobin: Target DNAs hybridized with 5′-blocking DNA were immobilized on a streptavidin-coated 96-well plate via the 3′-probe, then chemiluminescence was detected after incubation with myoglobin and target DNAs. Gray arrows indicate the experimental workflow, and the black arrows indicate the binding of myoglobin; the solid arrow represents strong binding, while the dashed arrow indicates weak binding. (**b**) Chemiluminescence intensity measurements of 200 nM unmethylated/methylated BCL2-87 and polyT 87. The statistical significance in (**b**) was determined via a one-way ANOVA followed via Tukey’s multiple comparisons test (*: *p* < 0.05; **: *p* < 0.01; ***: *p* < 0.001). (**c**) Chemiluminescence intensity measurements of unmethylated/methylated BCL2-87 at varying DNA sample concentrations. The statistical significance in (**c**) was determined using the multiple unpaired *t*-test. (*: *p* < 0.05; **: *p* < 0.01). All plots and bars are shown as the mean ± SD on the graph (*n* = 3).

CpG methylation in the target DNA containing the G4-forming sequence was detected. Previously, we detected CpG methylation by immobilizing the directly biotinylated target sequence, which lacked the versatility required for analyzing complex samples such as genomic DNA. Therefore, we shifted to a strategy that specifically captures the target sequence from the solution. Specifically, BCL2-87, which is an 87-mer DNA including the BCL2 G4 portion of the BCL2-G4 sequence, was used as the target sequence (Table S1). A key component of our revised approach involved using DNA probes complementary to the 3′-end of BCL2-87, which was then immobilized onto a streptavidin-coated 96-well plate (Table S1). This probe immobilization strategy is crucial because it enables the specific capture of the target BCL2-87, particularly from processed samples such as genomic DNA digested with restriction enzymes, thereby facilitating the subsequent methylation analysis (Figure 4a).

As shown in Figure 4b, when 200 nM BCL2-87 was used, the chemiluminescence intensity of the methylated BCL2-87 seq was significantly higher than that of the unmethylated BCL2-87. Moreover, the chemiluminescence intensity increased in a DNA-concentration-dependent manner in methylated and unmethylated BCL2-87 (Figure 4c). At a target DNA concentration of more than 25 nM, the chemiluminescence intensities of methylated BCL2-87 were significantly higher than those of unmethylated BCL2-87. The mean intensity increased from ~5.9 × 10^4^ a.u. (SD = 4.6 × 10^3^ a.u.) for 0% methylation to 7.9 × 10^4^ a.u. (SD = 1.1 × 10^3^ a.u.) for 100% methylation. When the chemiluminescence intensity and the methylation level were linearly related, the average sensitivity was a signal intensity change of ~195 a.u. for 1% methylation. Considering the sensitivity and measurement variability (represented by a pool SD of ~3.5 × 10^3^ a.u.), our analysis indicates that this method can distinguish a more than 50% difference in methylation level based on the 3σ criterion. This result is comparable to that obtained using the direct immobilization method.

To evaluate the potential of this method for practical applications, we assessed its performance in human serum, a complex biological matrix. Unmethylated and methylated BCL2-87 samples were spiked into serum at a final concentration of 200 nM. Consistent with the result obtained in buffer, methylated BCL2-87 exhibited a significantly higher chemiluminescence intensity than the unmethylated sample, even within the complex serum matrix (Figure S4). This finding demonstrates the robustness of our assay system using myoglobin against potential interferents in serum and supports its feasibility for the analysis of clinical samples in the future.

### 3.4. Investigation of the Structure-Forming DRD2 Seq

**Figure 5:**
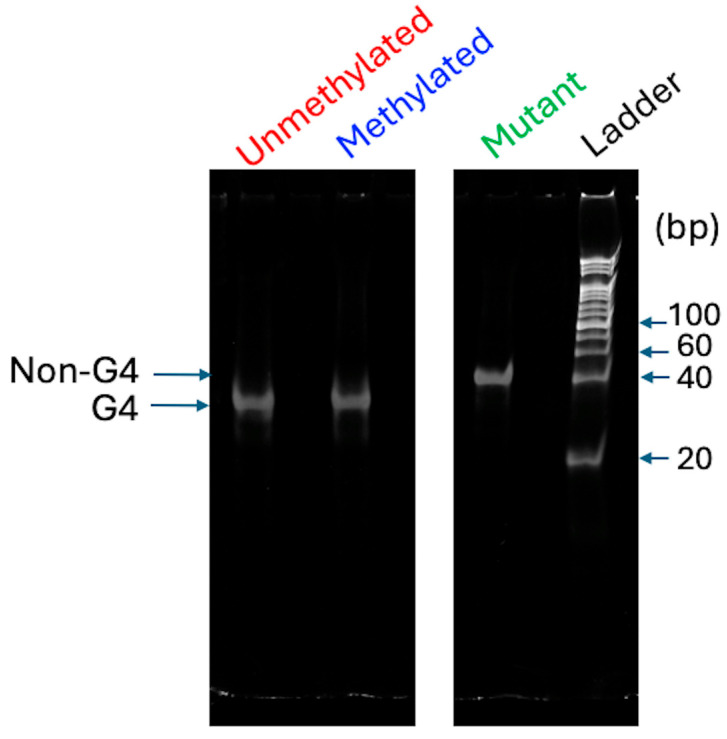
Electrophoretic analysis of 2 μM unmethylated, methylated, and mutant DRD2 seq. The DNA oligonucleotides were electrophoresed in 20% polyacrylamide gel.

To establish the broader applicability and versatility of our detection platform, we extended our analysis to a distinct G4-forming candidate sequence (DRD2 seq) from the promoter of the DRD2 gene (hg38, chr11: 113475566–113475620). This target was specifically chosen for its clinical relevance, given its previously reported association with schizophrenia [11]. The selected DRD2 seq is characterized by the presence of multiple G-tracts, which strongly predict its capacity to form the G4 structure, thereby serving as a test case for demonstrating the versatility of our method. First, to investigate the molecular properties and conformational diversity, we performed native PAGE analysis. In this analysis, a mutant DRD2 seq was used as the control, which was also designed to be deficient in G4 formation by altering a GGG motif to GTG within the unmethylated DRD2 seq, similar to mutant BCL2-G4 (Table S1). As shown in Figure 5, although the unmethylated/methylated and mutant DRD2 seq all have the same base lengths, bands corresponding to both the unmethylated and methylated DRD2 seq exhibited an increased electrophoretic mobility compared with that of the mutant DRD2 seq. This is consistent with the results of previous structural analyses and evaluations of the G4 structure [48,49]. Thus, the increased mobility was consistent with both unmethylated and methylated DRD2 seq forming compact G4 structures.

**Figure 6:**
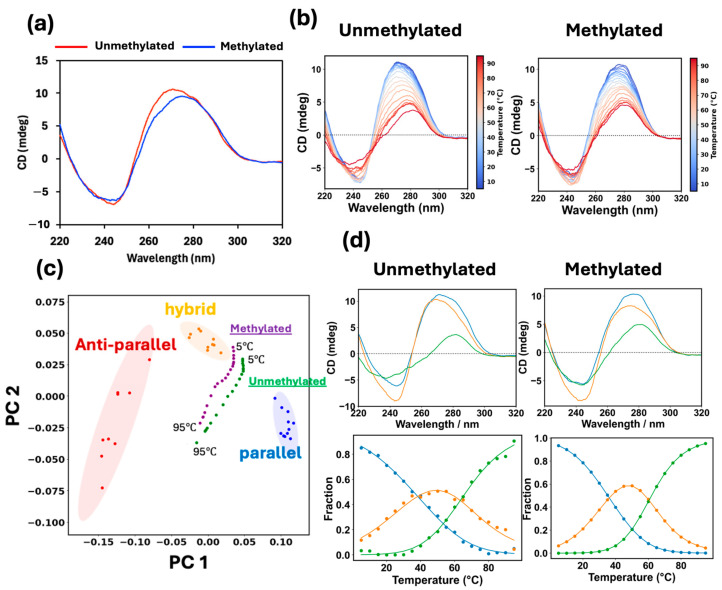
(**a**) CD spectra of 20 μM unmethylated (red line) and methylated DRD2 seq (blue line). (**b**) Temperature-dependent CD spectra of 20 μM unmethylated (left panel) and methylated DRD2 (right panel). The results shown in blue are the data obtained at low temperatures, while those shown in red represent the data obtained at high temperatures. (**c**) The PCA score plot and the colored areas indicate the 95% confidence interval of each topology of aptamers. The green and purple circles represent the unmethylated and methylated DRD2 seq results, respectively. (**d**) Results of spectral deconvolution by mcMCR analysis of unmethylated (left panels) and methylated (right panels) DRD2 seq. The pure spectra are shown in the upper panels. The ratio of each topology analyzed for the pure spectra is plotted in the bottom panels. The blue, orange, and green lines correspond to pure spectra 1,2, and 3, respectively.

To characterize the topology of G4 formed by DRD2 seq and to investigate the impact of methylation on the G4 topology, CD measurements were also performed. As shown in Figure 6a, the unmethylated DRD2 seq gave rise to a negative peak at ~240 nm and a positive peak at ~270 nm, while the methylated DRD2 seq produced a negative peak at ~240 nm and a positive peak at ~275 nm. These peaks do not seem to be attributed to the G4 structure. Therefore, to further analyze the topology, PCA [46] and spectral deconvolution by model constraint multivariate curve resolution (mcMCR) were performed [47].

The results of CD spectral analysis and PCA represent the topological changes with temperature (Figure 6b,c). Notably, PCA score plots revealed that the data points for methylated DRD2 seq clustered near the region characteristic of hybrid G4 (Figure 6c). Conversely, data points for the unmethylated DRD2 seq were localized away from the area of hybrid G4 compared with methylated DRD2, indicating a different structural response to methylation (Figure 6c).

The results of the mcMCR analysis visualize the topological changes with temperature, and the number of components can be estimated during the transition. The analysis of the unmethylated DRD2 seq revealed the formation of two different types of G4 structures at 25 °C (Figure 6d, bottom left panel). The first type, characterized by its pure orange spectrum (Figure 6d, top left panel), gave rise to a negative peak at 240 nm and a positive peak at ~260 nm in the CD spectrum, indicating a parallel G4 structure [46]. The second type, characterized by its pure blue spectrum (Figure 6d, top left panel), produced a negative peak at 240 nm, a positive peak at ~270 nm, and a distinct shoulder at 280 nm, which are characteristics of a hybrid G4 topology [50]. These findings demonstrated that unmethylated DRD2 seq coexists as a mixture of parallel and hybrid G4 structures at 25 °C.

Similar to its unmethylated analog, the methylated DRD2 seq also formed two different types of G4 structures (Figure 6d, bottom right panel). The first type, characterized by its pure orange spectrum (Figure 6d, right top panel), produced a CD spectrum with a negative peak at 240 nm, a positive peak at ~270 nm, and a shoulder at 290 nm, consistent with a hybrid G4 structure [46]. The second type, characterized by its pure blue spectrum (Figure 6d, top right panel), gave rise to a negative peak at 240 nm, a positive peak at ~270 nm, and a distinct shoulder at 280 nm, indicating a different hybrid G4 topology [51]. These findings demonstrated that the methylated DRD2 seq coexists as a mixture of different hybrid G4 structures at 25 °C. Notably, the unmethylated DRD2 seq exists as both parallel and hybrid G4 structures, whereas the methylated DRD2 seq forms two different types of hybrid G4 structures at 25 °C. Thus, CpG methylation affects not only the predominant G4 topology but also the heterogeneity of the G4 structural ensemble.

### 3.5. Investigation of the Interaction Between DRD2 Seq and Myoglobin, and Detection of CpG Methylation in DRD2 Seq Based on This Interaction

**Figure 7:**
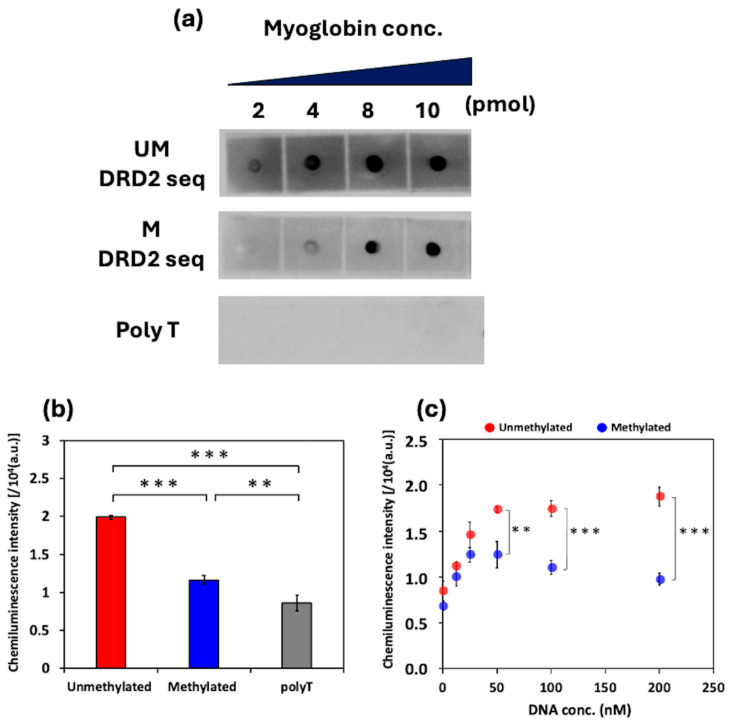
(**a**) Results of binding ability analysis by dot blotting of 100 nM unmethylated/methylated DRD2 seq and polyT. PolyT was used as a negative control, as it does not form a G4 structure. UM: unmethylated; M: methylated. (**b**) Chemiluminescence intensity measurements of 200 nM unmethylated/methylated DRD2 seq and polyT. (**c**) Chemiluminescence intensity measurements of unmethylated/methylated DRD2 seq at varying DNA sample concentrations. Statistical significances in (b) were determined using a one-way ANOVA followed by Tukey’s multiple comparisons test (**: *p* < 0.01, ***: *p* < 0.001). Statistical significance in (**c**) was determined using a multiple unpaired *t*-test. (**: *p* < 0.01, ***: *p* < 0.001). All plots and bars are shown as the mean ± SD on the graph. (*n* = 3).

To investigate the binding interaction between myoglobin and the DRD2 seq, a dot blotting assay was performed. The results are shown in Figure 7a. The spot signal intensity increased depending on the immobilized myoglobin concentration, indicating that both the unmethylated and methylated DRD2 seq were bound to myoglobin. However, the spot signal intensities of the unmethylated and methylated DRD2 seq at low myoglobin concentrations differed. The spot signal intensities were analyzed using ImageJ software, and at 2 μM myoglobin, the unmethylated DRD2 seq exhibited a higher spot signal intensity (150 a.u.) than methylated DRD2 seq, which exhibited an intensity of 80 a.u. Similarly, at 4 μM myoglobin, the intensities were 190 a.u. for unmethylated DRD2 seq and 110 a.u. for methylated DRD2 seq. These results indicate that the unmethylated DRD2 seq had a stronger binding affinity for myoglobin than methylated DRD2 seq. Our results reveal a contrasting effect of methylation on myoglobin binding: the binding affinity for DRD2 seq decreases upon methylation, while that for BCL2-G4 increases (Figure 2). Correlating these opposing results with their respective G4 structural changes indicates that the parallel G4 topology is the preferred structure for myoglobin binding.

Next, DRD2 seq methylation was detected on the basis of changes in myoglobin-derived peroxidase-like activity. As shown in Figure 7b, when DRD2 seq samples at a concentration of 200 nM were used, the chemiluminescence intensity of the unmethylated DRD2 seq was significantly higher than that of the methylated DRD2 seq. The mean intensity decreased from ~2.0 × 10^4^ a.u. (SD = 270 a.u.) for 0% methylation to 1.2 × 10^4^ a.u. (SD = 580 a.u.) for 100% methylation. When the chemiluminescence intensity and methylation level were linearly related, the average sensitivity corresponded to a signal intensity change of ~83 a.u. for 1% methylation. Considering this sensitivity alongside the measurement variability (represented by a pooled SD of ~450 a.u.), our analysis results indicate that this method can distinguish differences of >20% in DRD2 seq methylation based on the 3σ criterion. In Figure 7c, the chemiluminescence intensity increases in a DNA-concentration-dependent manner for the methylated and unmethylated DRD2 seq. At target DNA concentrations above 50 nM, the chemiluminescence intensities of the unmethylated DRD2 seq were significantly higher than those of the methylated DRD2 seq. The minimal DNA concentration required for methylation detection would differ depending on the DNA sequence. These results indicate the successful application of this assay to two distinct and clinically relevant G4-forming sequences, demonstrating the versatility of our approach as well as its potential for broader application to other G4-forming targets.

## 4. Conclusions

In this study, we focused on the structural change in G4 induced by CpG methylation and the enhancement of G4 activity with respect to the peroxidase-like activity of myoglobin. Our results suggested that the structural alteration shifts the binding mode of G4 to myoglobin, thereby modulating the binding affinity and the catalytic activity of the myoglobin-G4 complex. We established a detection method for CpG methylation in G4-forming sequences using myoglobin. Although this method is only effective for the sequences that can form G4 structures, CpG methylation usually occurs in G-rich sequence regions, where the formation of G4 structures is highly probable. Our method can provide a simple and straightforward analytical workflow that reduces the multistep complexities often associated with methods such as bisulfite conversion and sequencing. Notably, it does not require the complex synthesis of specialized G4 ligands. The measurement of the intensity of chemiluminescence catalyzed by myoglobin for the target DNA enables the detection of methylation levels. Additionally, because probes can be designed specifically for target DNAs, the methylation of multiple target DNAs can be simultaneously analyzed using myoglobin and microarrays with DNA probes.

However, translating this proof of concept into practical application faces two major challenges: first, the efficient preparation of the target ssDNA from complex samples such as genomic DNA, and second, the need to improve the assay’s resolution and sensitivity. Our data demonstrated that the method can reliably distinguish a large difference in methylation (~50% or greater). This level of resolution may be sufficient for specific applications, such as the diagnosis of certain congenital diseases known to exhibit dramatic changes in methylation. However, a key challenge is to improve this resolution to enable the detection of more subtle methylation differences, which are relevant in cancer and mental illness. Compounding this, the overall sensitivity must be enhanced. The current nanomolar detection limit is insufficient for many clinical applications that require the detection of low-abundance target DNAs, such as ~10^7^ copies (16.6 fM) of a specific sequence typically extracted from leukocytes in 1 mL of peripheral blood [52]. Apart from these technical challenges, it should be noted that chemiluminescence intensity and dynamic range depend on the target G4 sequence and assay configuration, including the probe-based assay. Therefore, establishing a calibration curve of each target sequence and assay configuration is necessary for accurate quantification.

Overcoming these challenges is crucial for future development. The issue of ssDNA accessibility could be addressed by further optimizing sample preparation protocols. To solve the sensitivity challenge, a particularly promising strategy involves empirically enhancing the core catalytic system, which is a logical approach given that the precise molecular mechanism of G4-mediated myoglobin activation remains to be fully clarified. This strategy could encompass protein engineering to create myoglobin variants with superior G4-dependent peroxidase-like activity, as well as the systematic optimization of chemiluminescence substrates and reaction kinetics to maximize signal output. The successful integration of these advancements would elevate this novel assay from a proof of concept to a powerful, rapid, and straightforward platform, significantly broadening its utility in both fundamental epigenetic research and future clinical diagnostics.

## Data Availability

The original contributions presented in this study are included in the article/Supplementary Material. Further inquiries be directed to the corresponding authors.

## Supplementary Materials

The following supporting information can be downloaded at https://www.mdpi.com/article/10.3390/bios16010001/s1. Figure S1: (a) Circular dichroism spectra of 20 μM unmethylated and methylated BCL2-G4; (b) Plot after principal component analysis. Figure S2: Binding ability analysis via dot blotting of 100 nM unmethylated mutant, methylated mutant BCL2-G4, unmethylated BCL2-G4, and polyT. Figure S3: Chemiluminescence intensity measurement of 200 nM unmethylated BCL2-G4, unmethylated and methylated mutant BCL2-G4, and polyT. Figure S4: Chemiluminescence intensity measurements of 200 nM unmethylated and methylated BCL2-87, and no target DNA samples spiked with human serum to obtain a final concentration of 5%. Table S1: DNA sequences used in this study.
