# Supplementary material for: Detection of Structural Changes in G-Quadruplex-Forming DNA Oligonucleotides via DNA Methylation Based on Luminol Chemiluminescence Catalyzed by Myoglobin
Source: Biosensors (Basel). 2025 Dec 19;16(1):1. doi: 10.3390/bios16010001 (PMC12838553; doi:10.3390/bios16010001)
Supplement: Supplementary file 1 [file biosensors-16-00001-s001.zip › biosensors-3989036-supplementary.pdf]

Supplementary Material

Detection of Structural Changes in G-Quadruplex-Forming DNA Oligonucleotides via DNA Methylation Based on Luminol Chemiluminescence Catalyzed by Myoglobin

Shintaro Inaba, Haruka Kawai, Mizuki Tomizawa, Daimei Miura, Kaori Tsukakoshi,

Kazunori Ikebukuro*

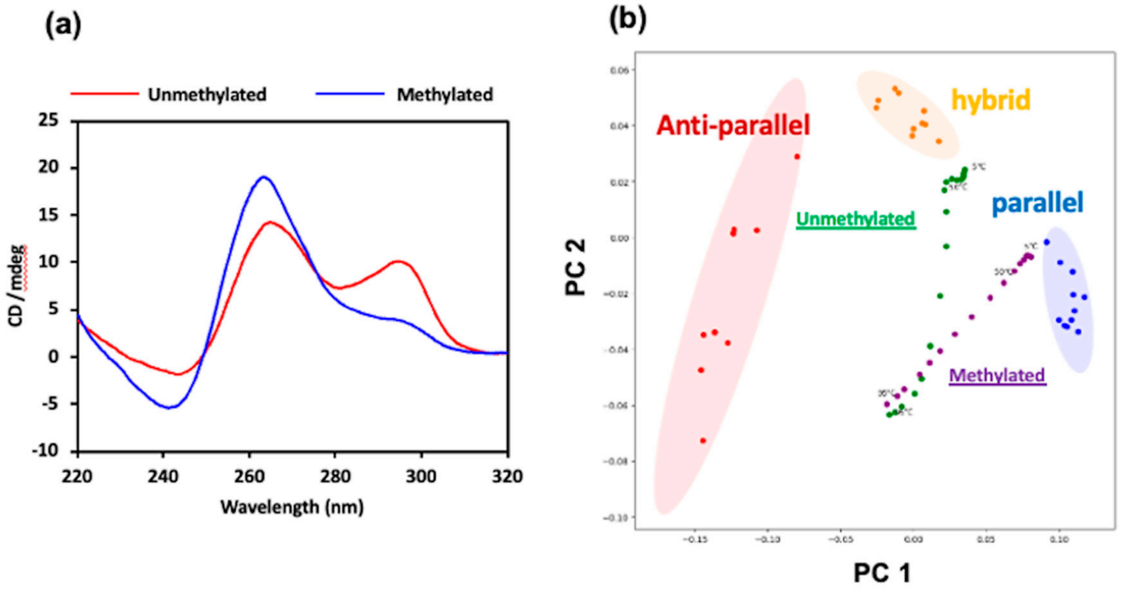

Figure S1. (a) Circular dichroism spectra of 20 μM unmethylated (red line) and methylated (blue line) BCL2-G4. (b) Plot after principal component analysis. The colored areas show the 95% confidence interval of each aptamer topology. The green and purple circles indicate the results of the unmethylated and methylated BCL2-G4, respectively.

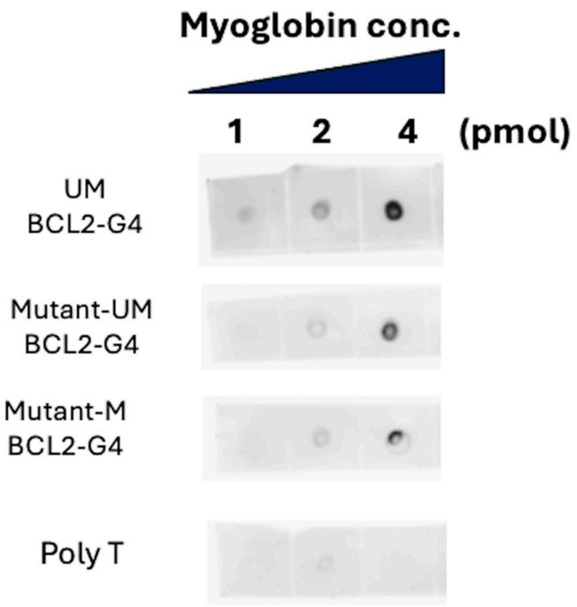

Figure S2. Binding affinity analysis by dot blotting of 100 nM unmethylated mutant, methylated mutant BCL2-G4, unmethylated BCL2-G4, and polyT. UM: unmethylated; M: methylated.

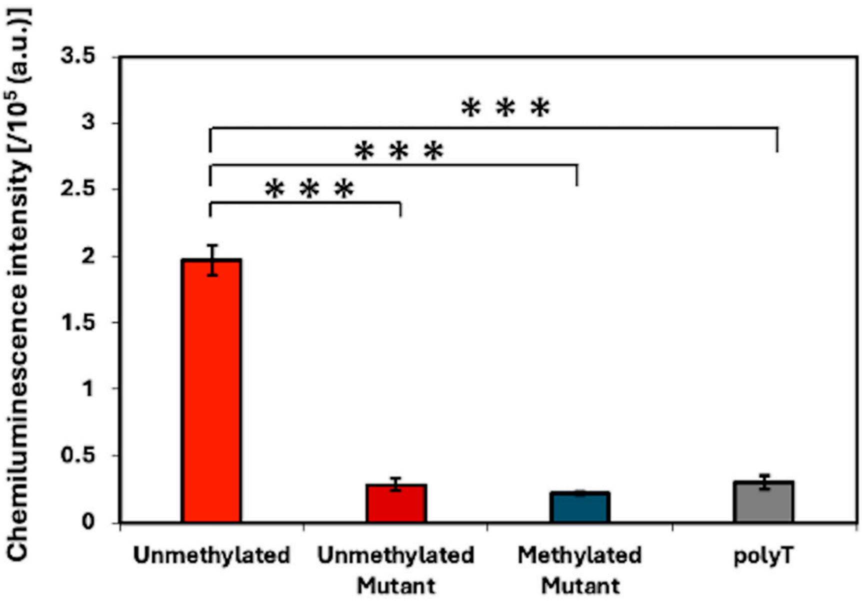

Figure S3. Chemiluminescence intensity measurements of 200 nM unmethylated BCL2-G4, unmethylated and methylated mutant BCL2-G4, and polyT. Statistical significance was determined using a one-way ANOVA, followed by Tukey's multiple comparisons test (***: $p < 0.001$).

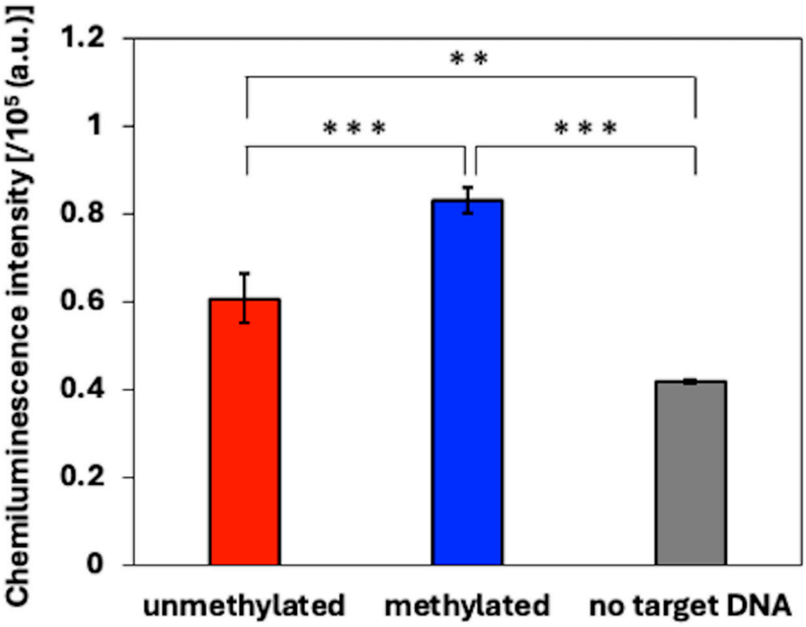

Figure S4. Chemiluminescence intensity measurements of 200 nM unmethylated and methylated BCL2-87, and no target DNA samples spiked with human serum to obtain a final concentration of 5%. Statistical significance was determined using a one-way ANOVA, followed by Tukey's multiple comparisons test (**: $p < 0.01$; ***: $p < 0.001$).

Table S1. DNA sequences used in this study. The methylated cytosine is shown in red, and the mutant site is shown in green.

Name	Sequence (5' to 3')	Length (mer)
BCL2-G4	CGGGCGCGGGAGGAAGGGGGCGGGAGC	27
Mutant BCL2-G4	CGTGCGCGTGAGGAAGTGTGCGTGAGC	27
polyT	TTTTTTTTTTTTTTTTTTTTTTTTTTT	27
BCL2 87	GCGGGCGGGCGGGCAGGCGGCGCGGAGGGGCGG GCGCGGGAGGAAGGGGGCGGGAGCGGGGCTGTG GTGCCTGTCCTCTTACTTCAT	87
5'-blocking BCL2-87	CCCCTCCGCGCCGCCTGCCCGCCCGCCCGC	30
3'-probe BCL2-87	ATGAAGTAAGAGGACAGGCACCACAGCCCC	30
polyT 87	GCGGGCGGCGGGCAGGCGGCGCGGAGGGGTTTTT TTTTTTTTTTTTTTTTTTTTTTTGGGGCTGTGGTGCC TGTCCTCTTACTTCAT	87
DRD2 seq	GGGTGGGAGCGCAGTGGGCGGTCGAGGGTTGCGT TCCCGCCTCAAAACAAGGGA	54
Mutant DRD2 seq	GTGTGTGAGCGCAGTGTGCGGTCGAGTGTTGCGT TCCCGCCTCAAAACAAGTGA	54
